# Supplementary material for: Genetic deficiency of NOD2 confers resistance to invasive aspergillosis
Source: Nat Commun. 2018 Jul 6;9:2636. doi: 10.1038/s41467-018-04912-3 (PMC6035256; doi:10.1038/s41467-018-04912-3)
Supplement: Supplementary file 1 — Supplementary Information [file 41467_2018_4912_MOESM1_ESM.pdf]

Supplementary materials

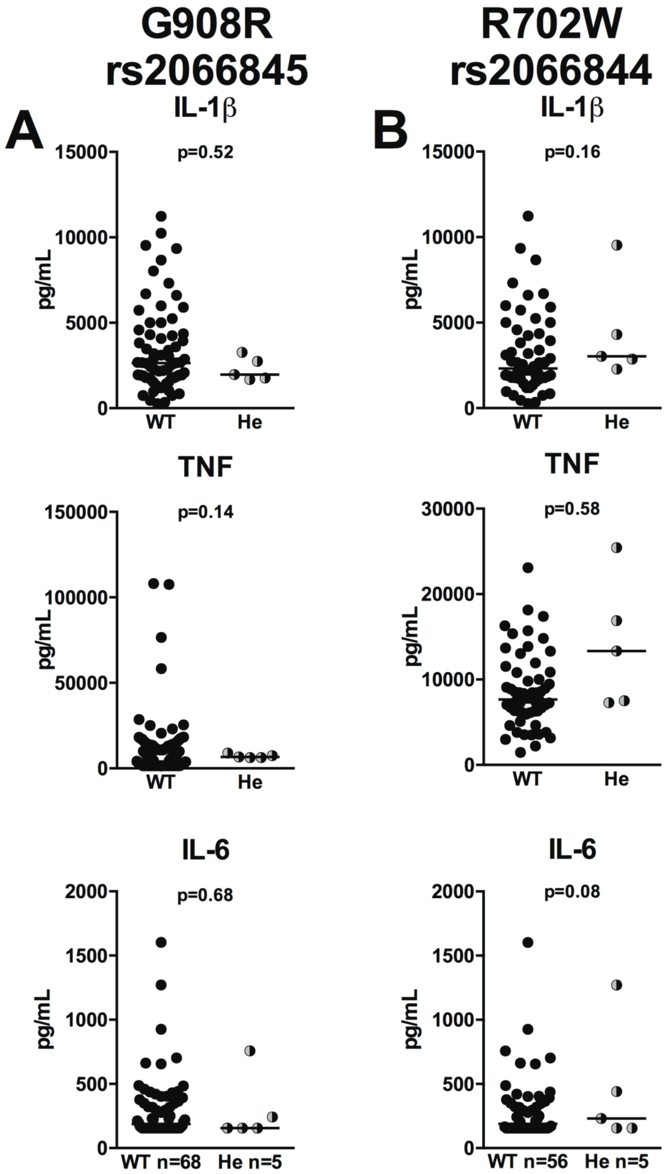

Supplementary Figure 1: influence of G908R and R702W *NOD2* polymorphisms on *Aspergillus*-induced IL-1β, TNFα and IL-6 release

IL-1β, TNF and IL-6 levels measured in culture supernatants of PBMCs stimulated with live *Aspergillus* conidia for 24 hours. The PBMCs of individuals with various genotypes of the *Nod2* receptor were compared. These genotypes included (A) the G908R mutation (wild-type; WT n=68; black circles and heterozygous; He n=5; half filled circles) and (B) the R702W mutation (wild-type; WT n=56; black circles, heterozygous; He n=5; half filled circles). Data is presented as scatterplots with a line indicating the median. The means of both groups were compared for significance using the Mann Whitney U test.

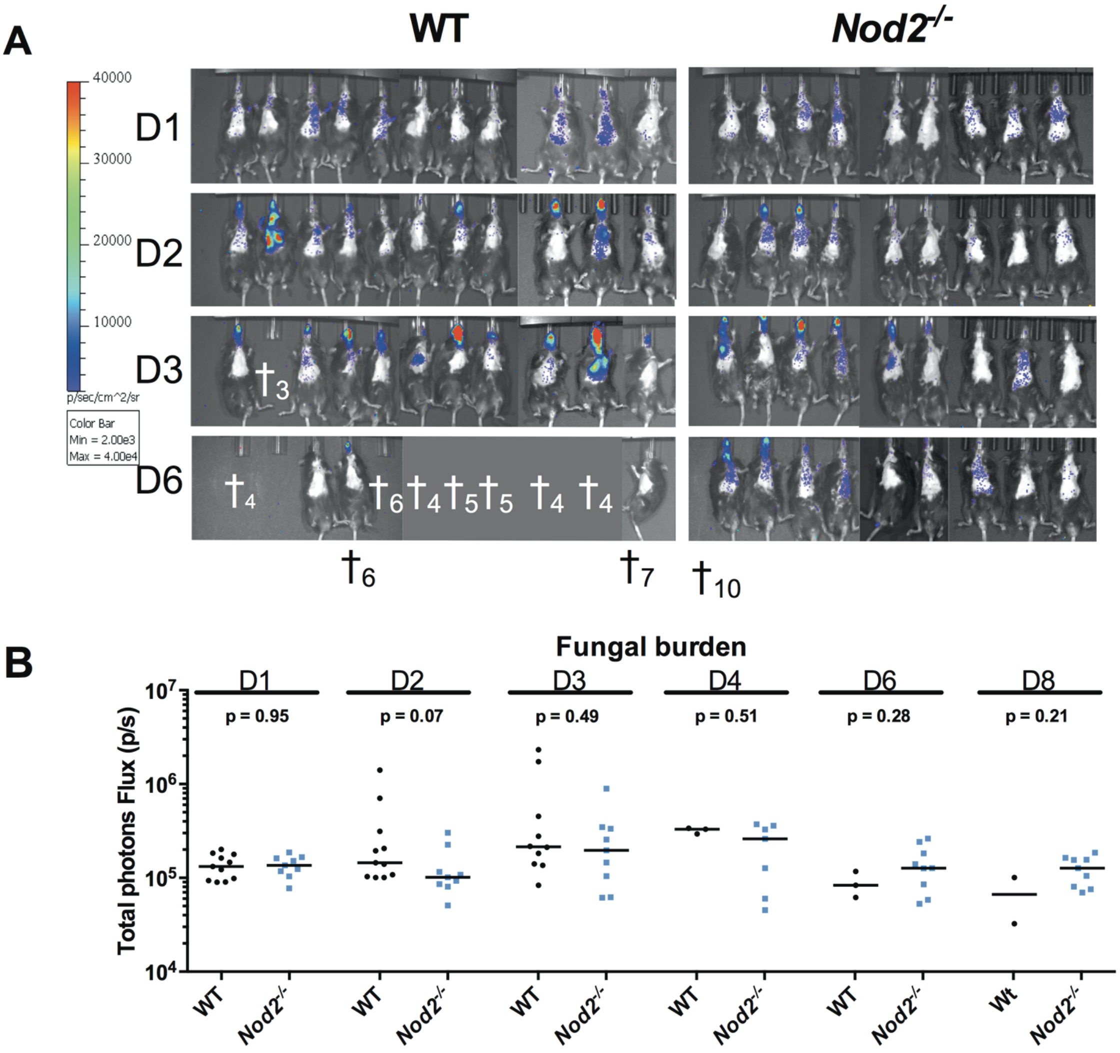

Supplementary Figure 2: fungal burden assessed by luminescence during survival

(A) Fungal burden assessed by luminescence signal at day 1, 2, 3 and 6 post infection from the luminescent *Aspergillus* originating from lung and sinus regions in WT (n=11) and *Nod2*$^{-/-}$ (n=9) mice. † indicating the day of death of the mice. (B) Scatterplots indicate measurements from individual mice with lines indicating the median value, black dots representing WT mice and blue squares representing *Nod2*$^{-/-}$ mice. For each time point means were compared for significance using the Mann Whitney U test.

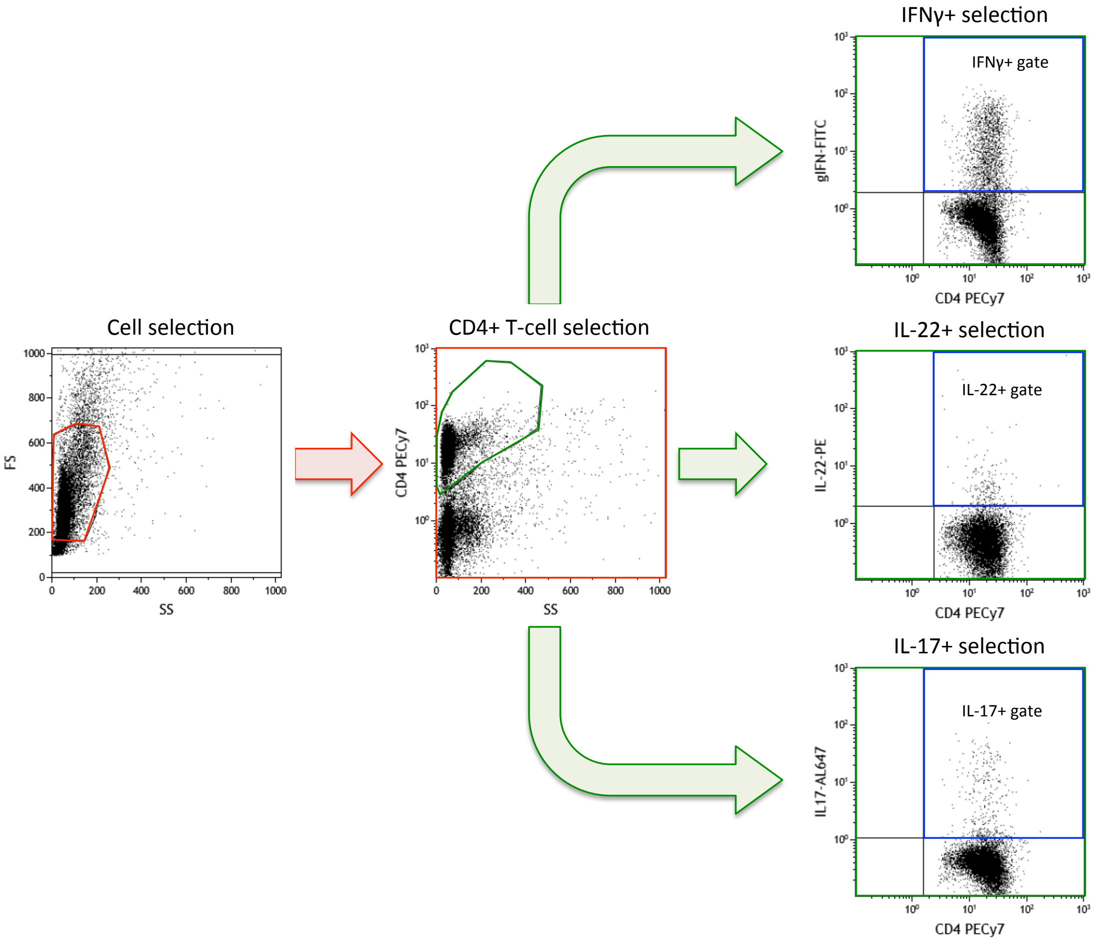

Supplementary Figure 3: gating strategy for T-cell subsets

All events were plotted based on forward scatter (FS) and side scatter (SS) characteristics. Within this plot the region of cells containing lymphocytes was gated (red gate) and examined for CD4 expression. Within the cell selection the CD4 PeCy7 signal was plotted against the side scatter (SS) and the population of CD4+ cells was gated (green gate). Within the CD4+T-cell selection IFNγ, IL-22, and IL-17A expression was examined by plotting gIFN-FITC, IL-22-PE and IL-17-Alexa647 signals respectively against the CD4 PeCy7 signal. The percentage of cells within the CD4+ population positive for the respective cytokines (top right quadrant; blue gates) were used for analysis.

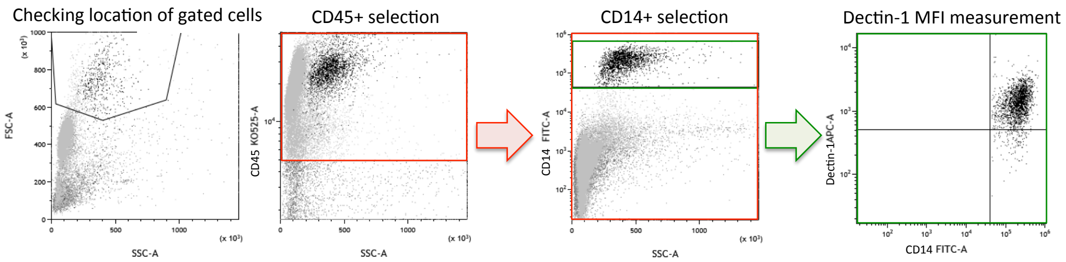

Supplementary Figure 4: gating strategy for surface dectin-1 expression

Monocytes or macrophages were plotted based on their forward scatter (FSC-A) and side scatter (SSC-A) characteristics. No cells were gated in this plot this was solely performed to check whether the gated cells correspond with the expected forward scatter and side scatter characteristics of monocytes or macrophages. First CD45+ leukocytes were gated (red gate) within a plot of the CD45 KromeOrange(KO525-A) signal against the side scatter characteristics (SSC-A). On this population of cells the CD14+ cells were gated (green gate) within a plot of the CD14 FITC (CD14 FITC-A) signal against the side scatter characteristics (SSC-A). Within the CD14+ selection gate the mean fluorescence intensity (MFI) of the Dectin-1 APC signal (Dectin-1 APC-A) was measured.

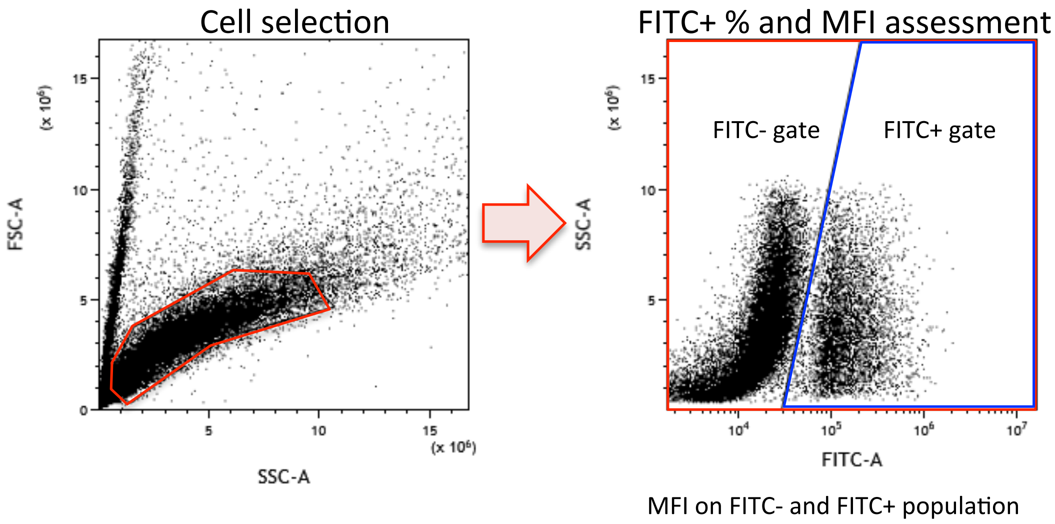

Supplementary Figure 5: gating strategy for *Aspergillus* phagocytosis

Macrophages were plotted based on their forward scatter (FSC-A) and side scatter (SSC-A) characteristics and within this plot the macrophages were gated (red gate). The selected cells were plotted for side scatter (SSC-A) characteristics against the FITC signal of *Aspergillus*. The percentage of FITC positive macrophages was determined using a FITC+ gate (blue gate) to determine the amount of monocytes phagocytosing and the mean fluorescence intensity was determined on the FITC+ macrophages (blue gate) to determine the relative amount of phagocytosed fluorescent conidia.

Supplementary Table 1 – LD analysis of the NOD2 SNPs evaluated in our study.

RefSNP	rs2066842	rs2066844	rs2066845	rs2066847
**rs2066842**	D'=1<br>r^2=1	D'=1<br>r^2=0.163	D'=1<br>r^2=0.044	D'=1<br>r^2=0.067
**rs2066844**	D'=1<br>r^2=0.163	D'=1<br>r^2=1	D'=1<br>r^2=0.002	D'=1<br>r^2=0.002
**rs2066845**	D'=1<br>r^2=0.044	D'=1<br>r^2=0.002	D'=1<br>r^2=1	D'=1<br>r^2=0.001
**rs2066847**	D'=1<br>r^2=0.067	D'=1<br>r^2=0.002	D'=1<br>r^2=0.001	D'=1<br>r^2=1

Supplementary Table 2 – Baseline characteristics of patients enrolled in the study.

Variables	IA (n=66) n (%)	No IA (n=244) n (%)	P value
**Age at transplantation, no (%)**			
≤20 years	10 (15.1)	45 (18.4)	0.45
21 – 40 years	17 (25.8)	77 (31.6)	
>40 years	39 (59.1)	122 (50.0)	
**Gender, no (%)**			
Female	27 (40.9)	90 (36.9)	0.52
Male	39 (59.1)	154 (63.1)	
**Underlying disease, no. (%)**			
Acute leukemia	35 (53.0)	127 (52.1)	0.03
Chronic lymphoproliferative diseases	8 (12.1)	58 (23.8)	
Chronic myeloproliferative diseases	4 (6.1)	13 (5.3)	
Myelodysplastic/myeloproliferative diseases	11 (16.7)	15 (6.1)	
Aplastic anemia	6 (9.1)	15 (6.1)	
Other	2 (3.0)	16 (6.6)	
**Transplantation type, no. (%)**			
Matched, related	18 (25.7)	104 (40.3)	0.04
Matched, unrelated	31 (44.3)	73 (28.3)	
Mismatched, related	0 (0.0)	5 (1.9)	
Mismatched, unrelated	21 (30.0)	76 (29.5)	
**Graft source, no. (%)**			
Peripheral blood	58 (87.9)	193 (79.1)	0.24
Bone-marrow	8 (12.1)	46 (18.9)	
Cord blood	0 (0.0)	5 (2.0)	
**Disease stage, no. (%)**			
First complete remission	35 (53.0)	128 (52.5)	0.99
Second or subsequent remission, or relapse	11 (16.7)	42 (17.2)	
Active disease	20 (30.3)	74 (30.3)	
**Conditioning regimen, no (%)**			
RIC	45 (68.2)	150 (61.5)	0.35
Myeloablative	21 (31.8)	94 (38.5)	
**CMV serostatus of donor and recipient, no. (%)**			
D-/R+ or D+/R+	57 (86.4)	216 (88.5)	0.60
D-/R- or D+/R-	9 (13.6)	28 (11.5)	
**Duration of neutropenia, mean days (range)†**	12 (8 – 26)	13 (6 – 30)	0.31
**Acute GVHD, no. (%)**			
No GVHD or grades I – II	47 (71.2)	209 (85.7)	0.008
Grades III – IV	19 (28.8)	35 (14.3)	
**Antifungal prophylaxis, no. (%)‡**			
Fluconazole	36 (54.5)	119 (48.8)	0.64
Posaconazole	19 (28.8)	73 (29.9)	
Other	5 (7.6)	16 (6.6)	
None	6 (9.1)	36 (14.7)	

RIC, reduced intensity conditioning; CMV, cytomegalovirus; D, donor; R, recipient; GVHD, graft-versus-host-disease. †Neutropenia was defined as $\leq 0.5\times10^9$ cells/L. ‡Other antifungals used in prophylaxis included voriconazole (n=10), liposomal amphotericin B (n=7), itraconazole (n=2), and caspofungin (n=2). P values were calculated by Fisher's exact probability t-test or Student's t-test for continuous variables.
